# Induction of Inflammation in Vascular Endothelial Cells by Metal Oxide Nanoparticles: Effect of Particle Composition

**DOI:** 10.1289/ehp.8497

**Published:** 2006-12-11

**Authors:** Andrea Gojova, Bing Guo, Rama S. Kota, John C. Rutledge, Ian M. Kennedy, Abdul I. Barakat

**Affiliations:** 1 Department of Mechanical and Aeronautical Engineering, University of California, Davis, Davis, California, USA; 2 Department of Mechanical Engineering, Texas A&M University, College Station, Texas, USA; 3 Department of Internal Medicine, University of California, Davis, Davis, California, USA

**Keywords:** air pollution, atherosclerosis, cardiovascular disease, endothelial cells, inflammation, nanoparticles, particulate matter

## Abstract

**Background:**

The mechanisms governing the correlation between exposure to ultrafine particles and the increased incidence of cardiovascular disease remain unknown. Ultrafine particles appear to cross the pulmonary epithelial barrier into the bloodstream, raising the possibility of direct contact with the vascular endothelium.

**Objectives:**

Because endothelial inflammation is critical for the development of cardiovascular pathology, we hypothesized that direct exposure of human aortic endothelial cells (HAECs) to ultrafine particles induces an inflammatory response and that this response depends on particle composition.

**Methods:**

To test the hypothesis, we incubated HAECs for 1–8 hr with different concentrations (0.001–50 μg/mL) of iron oxide (Fe_2_O_3_), yttrium oxide (Y_2_O_3_), and zinc oxide (ZnO) nanoparticles and subsequently measured mRNA and protein levels of the three inflammatory markers intra-cellular cell adhesion molecule-1, interleukin-8, and monocyte chemotactic protein-1. We also determined nanoparticle interactions with HAECs using inductively coupled plasma mass spectrometry and transmission electron microscopy.

**Results:**

Our data indicate that nanoparticle delivery to the HAEC surface and uptake within the cells correlate directly with particle concentration in the cell culture medium. All three types of nanoparticles are internalized into HAECs and are often found within intracellular vesicles. Fe_2_O_3_ nanoparticles fail to provoke an inflammatory response in HAECs at any of the concentrations tested; however, Y_2_O_3_ and ZnO nanoparticles elicit a pronounced inflammatory response above a threshold concentration of 10 μg/mL. At the highest concentration, ZnO nanoparticles are cytotoxic and lead to considerable cell death.

**Conclusions:**

These results demonstrate that inflammation in HAECs following acute exposure to metal oxide nanoparticles depends on particle composition.

Although recent epidemiologic studies have demonstrated a correlation between exposure to fine particulate matter in air pollution and an increased incidence of cardiovascular morbidity and mortality (Peters et al. 2001; Pope et al. 2004; Samet et al. 2000), the mechanisms behind this correlation remain largely unknown. More recently, ultrafine particles (< 100 nm) have been reported to be particularly relevant pathologically because of their small size and high reactivity. Of particular relevance to the present study, ultrafine particles have been shown to cross the pulmonary epithelial barrier into the bloodstream (Kreyling et al. 2002; Nemmar et al. 2002, 2003), thereby directly exposing the vascular endothelium to particles.

Atherosclerosis is a primary cause of many cardiovascular complications including myocardial infarctions, ischemia, and stroke. Acute and chronic inflammation of the endothelium plays a central role in the development of atherosclerosis (Libby 2002; Ross 1999). We hypothesized that exposure of human aortic endothelial cells (HAECs) to metal oxide ultrafine particles elicits an inflammatory response and that the nature of the response depends on the composition of the particles. To test this hypothesis, we exposed cultured HAECs acutely to a wide range of concentrations of iron oxide (Fe_2_O_3_), yttrium oxide (Y_2_O_3_), and zinc oxide (ZnO) nanoparticles for periods of 1–8 hr, and subsequently assessed the impact of this exposure on mRNA and protein levels of specific inflammatory markers. We also used various imaging and spectrometry techniques to probe particle interactions with cells. Although all three types of nanoparticles interact with the cell surface and are ultimately uptaken into the intracellular space, induction of the inflammatory response depends on nanoparticle composition.

## Materials and Methods

### Synthesis of metal oxide nanoparticles

Fe_2_O_3_ nanoparticles were synthesized in an H_2_/air diffusion flame seeded with the vapor of Fe(CO)_5_ (99.5%; Alfa Aesar, Ward Hill, MA) using the system described by Guo and Kennedy (Guo and Kennedy 2004). The Fe(CO)_5_ (Fe pentacarbonyl) vapor decomposed in the flame to form Fe_2_O_3_ nanoparticles. Y_2_O_3_ nanoparticles were synthesized in a similar manner with the vapor of tris(2,2,6,6-tetramethyl-3,5-heptanedionato)Y(III) (98%; Alfa Aesar). The yttrium precursor, which sublimed at moderately high temperature, was placed in a stainless steel basket in a furnace and produced a vapor upon heating. ZnO nanoparticles were synthesized in a similar H_2_/air diffusion flame seeded with the vapor of metal Zn. Zn shots (99.999%; Alfa Aesar) were placed in a stainless steel furnace that was heated to approximately 600°C. The post-flame aerosol containing the particles was drawn into a sampling tube by vacuum and the particles were captured on a filter.

### Characterization of nanoparticles

The metal oxide nanoparticles were characterized using transmission electron microscopy (TEM), X-ray diffraction (XRD), and the Brunauer-Emmett-Teller (BET) technique. For the TEM and XRD analyses, the nanoparticles were first extracted from the membrane filter and suspended in ethanol. To prepare an XRD sample, the ethanol suspension containing the metal oxide particles was deposited drop-wise on a single crystal silicon substrate and dried to obtain a thin layer of the particles. The XRD samples were then analyzed in a Scintag PAD V X-Ray Diffractometer (Thermo Optek, Franklin, MA) with a Cu K_alpha_ radiation source operated at 45 kV and 40 mA. The XRD patterns were analyzed using the MDI JADE 6.0 program (Materials Data Inc., Livermore, CA). To prepare TEM samples, drops of the ethanol suspension were put on copper TEM grids with carbon film support. After evaporation of the solvent, the metal oxide nanoparticles were deposited on the grids. The TEM samples were then analyzed using a Philips CM-12 microscope (FEI, Hillsboro, OR) operated at 100 kV. The BET analysis was conducted on a Gemini 2360 instrument (Micromeritics, Norcross, GA). Each sample contained 50- to 100-mg nanoparticles. The sample was first degassed at 300°C to reach a pressure < 10 μm Hg to remove adsorbed water; the water content was determined gravimetrically. The specific surface area of the nanoparticle samples was then measured by N_2_ adsorption/desorption.

### Cell culture and exposure to nanoparticles

HAECs (Cascade Biologics, Portland, OR) in passages 6–8 were cultured using standard procedures in Medium 200 (Cascade Biologics) containing 100 U/mL penicillin G, 100 μg/mL streptomycin, 0.25 μg/mL amphotericin B, and Low Serum Growth Supplement (LSGS) (Cascade Biologics). LSGS supplementation resulted in medium containing 2% fetal bovine serum, 1 μg/mL hydrocortisone, 10 ng/mL human epidermal growth factor, 3 ng/mL basic fibroblast growth factor, and 10 μg/mL heparin. In the nanoparticle experiments, confluent HAEC monolayers in standard 6-well plates were incubated for 1-8 hr at 37°C with Fe_2_O_3_, Y_2_O_3_, or ZnO nanoparticles in cell culture medium at concentrations ranging from 0.001 to 50 μg/mL. Prior to dilution, nanoparticle stock solutions were sonicated for 5 min to break up aggregates. HAECs incubated with nanoparticle-free medium served as controls.

To determine the impact of nanoparticles on HAEC viability, cells that were incubated with the nanoparticles for 4 hr were washed twice with complete phosphate-buffered saline (PBS; Invitrogen, Carlsbad, CA) and then subjected to the trypan blue exclusion assay. To assess both short- and longer-term impact of nanoparticle exposure on HAEC viability, the trypan blue assay was performed immediately after the 4-hr incubation period as well as 24 hr after the end of incubation (during which time the cells were incubated in nanoparticle-free cell culture medium).

### Isolation of RNA and reverse transcription

Immediately following incubation with nanoparticles, HAECs were washed with PBS, detached by trypsinization, and collected by centrifugation. Total RNA was isolated using a guanidine isothiocyanate/phenol (TRIzol Reagent; Invitrogen) and chloroform-based isolation process in accordance with the manufacturer’s instructions. The RNA was subsequently precipitated by isopropanol, and the final pellet was resuspended in DEPC (diethylpyrocarbonate) water and stored at –80°C. cDNAs were prepared from 1 μg total RNA using the reverse transcriptase enzyme SuperScript II (Invitrogen) according to the manufacturer’s instructions.

### Quantitative real-time polymerase chain reaction (PCR) analysis

We probed transcription levels of three inflammatory markers: intracellular cell adhesion molecule-1 (ICAM-1), monocyte chemotactic protein-1 (MCP-1), and interleukin-8 (IL-8). Human glyceraldehyde-3-phosphate dehydrogenase (GAPDH) was used as an internal control. Primers for these markers were designed by Primer Express (Applied Biosystems, Foster City, CA) and purchased from Operon Technologies (Alameda, CA). Their sequences were as follows:

ICAM-1 sense:

5′CAGAAGAAGTGGCCCTCCATAG-3;

ICAM-1 antisense:

5′GGGCCTTTGTGTTTTGATGCTA-3;

MCP-1 sense:

5′GCCAAGGAGATCTGTGCTGAC-3;

MCP-1 antisense:

5′CATGGAATCCTGAACCCACTTC-3;

IL-8 sense:

5′GTGTAAACATGACTTCCAAGCTGG-3;

IL-8 antisense:

5′GCACCTTCACACAGAGCTGC-3;

GAPDH sense:

5′CACCAACTGCTTAGCACCCC-3;

GAPDH antisense:

5′TGGTCATGAGTCCTTCCACG-3.

We conducted quantitative real-time PCR using the GeneAmp 7900HT Sequence Detection System (Applied Biosystems). The reaction was preformed in 96-well Optical Reaction Plates (Applied Biosystems) with 25 μL of reaction mixture in each well. The reaction mixture contained samples of cDNA diluted 1:10, 500 nM of respective sense and antisense primers, and SYBR Green PCR Master Mix (Applied Biosystems). The PCR reaction consisted of initial thermal activation at 95°C for 10 min and 40 cycles. Each cycle was as follows: 95°C for 15 sec; 60°C for 1 min. We verified PCR products by analysis of heat-dissociation curves and amplification plots. Quantitative values were acquired from linear regression of the PCR standard curve. Expression levels for inflammatory genes were then normalized to GAPDH levels for each sample.

### Western blot analysis

Immediately after nanoparticle incubation, HAECs were washed with PBS and then lysed using a lysis buffer containing M-PER Mammalian Protein Extraction reagent (Pierce, Rockford, IL), 2 mM sodium orthovanadate, 5 mM sodium fluoride, 1 mM phenylmethyl-sulfonylfluoride, 1% protease inhibitor cocktail, and 0.1% triton X-100. The cell lysates were then centrifuged at 14,000 × *g* for 5 min at 4°C, and SDS-PAGE was performed on a 7.5% Tris-HCl gel (Bio-Rad Laboratories, Hercules, CA). Proteins were then transferred onto a nitrocellulose membrane. The membrane was blocked with 5% blotto non-fat milk (Santa Cruz Biotechnology, Santa Cruz, CA) and incubated overnight at 4°C with rabbit polyclonal anti-ICAM-1 (Santa Cruz Biotechnology) and mouse monoclonal anti-β-actin (Sigma Chemical Company, St. Louis, MO) primary antibodies. After incubation with respective secondary antibodies conjugated with horseradish peroxidase (HRP), HRP was visualized using ECL Western Detection Reagent (Amersham, Pittsburgh, PA) and developed on a CL-XPosure film (Pierce). We performed protein analysis using Kodak 1D Image Analysis Software (Eastman Kodak Company, Rochester, NY). Band densities of ICAM-1 were normalized by the band density of β-actin, which served as an internal control.

### ELISA analysis

Immediately after the nanoparticle incubation period, we collected cell culture supernatants and centrifuged them at 16,000 × *g* for 5 min to remove cell debris and particles. We assayed MCP-1 protein concentrations in the supernatants using an ELISA kit (Quantikine Human MCP-1/CCL2 Immunoassay, R&D Systems, Minneapolis, MN) according to the manufacturer’s instructions.

### TEM

HAECs exposed to nanoparticles for 4 hr were washed with PBS and fixed with Karnovsky’s EM fixative (2.5% glutaraldehyde and 2% paraformaldehyde in 80 mM phosphate buffer, pH 7.3–7.4). Secondary fixation was done in 1% osmium tetroxide with 1.5% potassium ferrocyanide in double distilled H_2_O for 1 hr at 4°C. Dehydration was through ascending concentrations of ethanol with three changes at 100%. Pure Epon-Araldite resin that did not contain methyl anhydride was added and infiltrated overnight at room temperature. All resin was removed the next day, and fresh resin was added to the appropriate depth. The sample was polymerized for 18 hr. Ultrathin sections of cells of interest were cut *en face* (parallel to the surface on which the cells were grown) using a Leica Ultracut UCT ultramicrotome (Leica Mikrosysteme Gmbh, Vienna, Austria) and then stained with uranyl acetate and lead citrate before viewing in a Philips CM120 electron microscope (FEI).

### Inductively coupled plasma mass spectrometry

We measured the delivery of the metal oxide nanoparticles to the cell surface and the uptake of nanoparticles by the cells using inductively coupled plasma mass spectrometry (ICP-MS). Confluent HAECs in 6-well plates were exposed to nanoparticles for 4 hr, washed with PBS, and detached. The cell pellet was resuspended in 0.5 mL Hanks’ Balanced Salt Solution (HBSS; Invitrogen), and the number of cells was established using a hemacytometer (Bright-Line; Hausser Scientific, Horsham, PA). The solutions were mixed with concentrated nitric acid (HNO_3_; EMD Chemicals, Gibbstown, NJ) to reach a final HNO_3_ concentration of 3%, and then heated to 80°C for 3 hr to dissolve cell content. We prepared a blank control solution for ICPMS reaction (without cells) by mixing 0.5 mL HBSS with the same amount of concentrated HNO_3_; this solution was processed the same way as the sample solutions. Finally, the dissolved solutions were adjusted to a volume of 25 mL with 3% HNO_3_ in water and used for ICP-MS analysis. The concentrations of Fe, Y, and Zn were determined using an Agilent Technologies 7500c inductively coupled plasma mass spectrometer (Agilent Technologies, Santa Clara, CA). The precision of the analysis was better than ± 3.8%.

### Statistical analysis

Data are presented as mean ± SE. Statistical analyses were performed by one-way analysis of variance followed by Dunnett post hoc test. Differences in means were considered significant if *p* < 0.05.

## Results

### Particle characteristics and uptake

**Figure 1:**
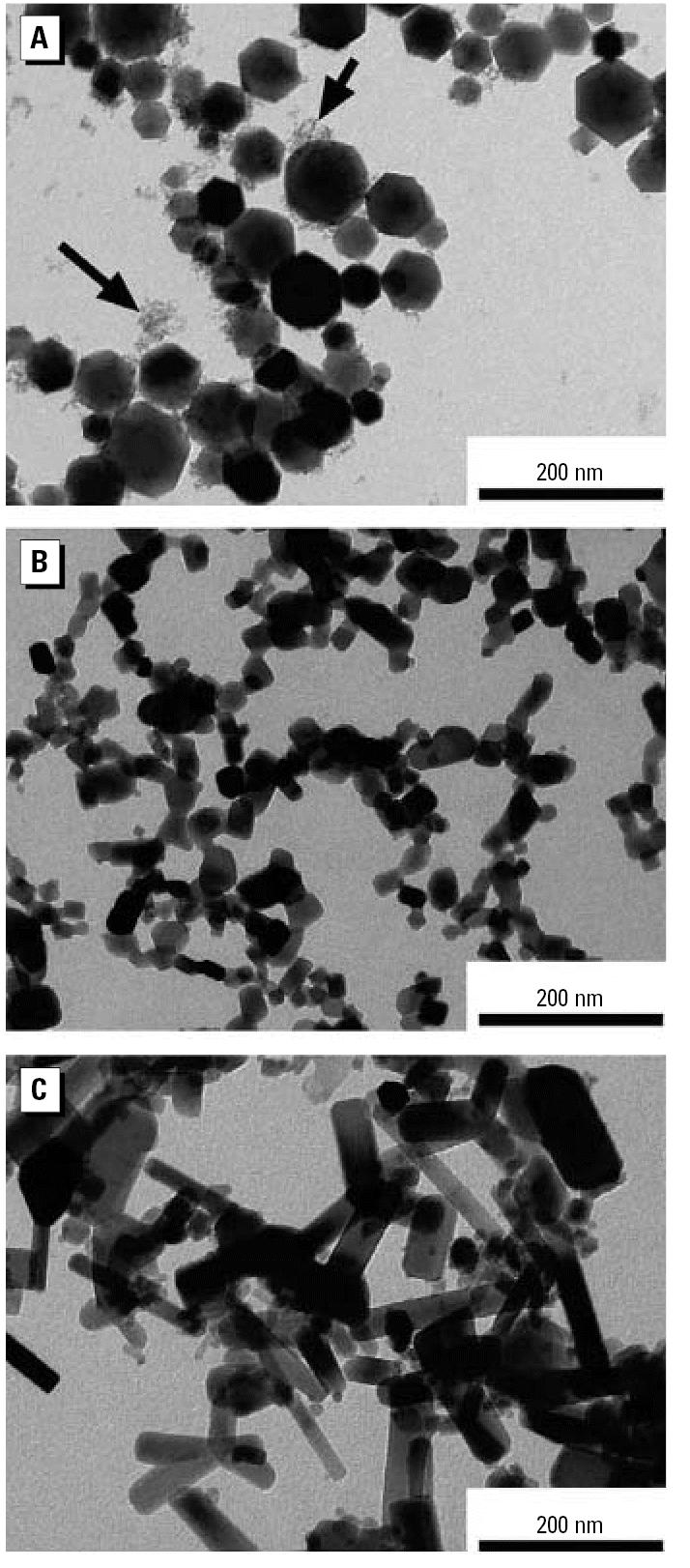
TEM images of metal oxide nanoparticles. (*A*) Fe_2_O_3_; arrows point to the subpopulation of particles with a size < 5 nm. (*B*) Y_2_O_3_. (*C*) ZnO.

Representative TEM images of the metal oxide nanoparticles are shown in Figure 1. XRD and TEM analysis revealed that the Fe_2_O_3_ nanoparticles exhibit the crystal structure of γ-Fe_2_O_3_, or maghemite, and can be divided into two size groups (Figure 1A). To further characterize the Fe_2_O_3_ particles, we used a Scanning Mobility Particle Sizer (SMPS; TSI Incorporated, Shoreview, MN), consisting of a 3071A Differential Mobility Analyzer (DMA) and a 3025A Condensation Particles Counter (CPC), for online particle size analysis. Consistent with the TEM images, the SMPS analysis demonstrated that Fe_2_O_3_ particles contain two size modes. Particles in the larger mode are nonagglomerated and nearly spherical, and appear to have a log-normal size distribution. Based on the SMPS and TEM surveys, the count median diameter (CMD) of the log-normal size distribution is approximately 45 nm, with a geometric standard deviation (GSD) of approximately 1.2. The particles in the small mode are nearly monodisperse with an estimated mean diameter of approximately 5 nm. Our SMPS could not resolve the small mode of the Fe_2_O_3_ particle size distribution and hence we resorted to the use of direct physical surface areas via the BET method, along with an analysis of the resolved size distribution of the larger particles to infer the contributions of each mode to number, surface area, and mass of the combined bimodal aerosol. The ratio of number concentration of the smaller mode to the larger mode can be calculated using the following equation:


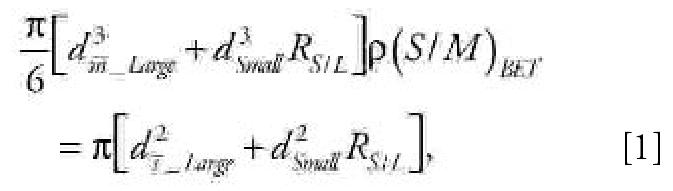


where *d_m_*_¯_*__Large_* is the diameter of particles with average mass for the large mode, which—calculated from the CMD and GSD of the assumed log-normal distribution (Hinds 1999)—is approximately 47 nm; *d_Small_* is the estimated diameter for the assumed monodisperse small mode, approximately 5 nm; *R*_ S/L_ is the number concentration ratio of the small mode to the large mode; (*S/M*)*_BET_* is the BET-specific surface area measured for the Fe_2_O_3_ particles, large and small modes both included, 81 ± 1 m^2^/g; and *d_s_*_¯_*__Large_* is the diameter of particles with average surface area for the large mode, which—calculated from the CMD and GSD of the log-normal distribution (Hinds 1999)—is approximately 47 nm. The equation can be solved to yield a value of approximately 330 for *R_S/L_*, indicating that for every particle in the large mode there are approximately 330 particles in the small mode. The results also indicate that the small mode accounts for approximately 28% of the particle mass and approximately 79% of the particle surface area.

The Y_2_O_3_ nanoparticles have a C-type cubic structure with primary particle sizes in the range of 20–60 nm (Figure 1B). The ZnO nanoparticles have a zincite crystal structure and are rod-shaped, with lengths of 100–200 nm and diameters of 20–70 nm (Figure 1C). The smaller size mode present in the case of Fe_2_O_3_ nanoparticles is not evident in the TEM images of the Y_2_O_3_ or ZnO particles. The particle size distribution of the Y_2_O_3_ and ZnO particles was not measured using the SMPS because of difficulty in diluting the flame-generated aerosols. The BET-specific surface areas for the Fe_2_O_3_, Y_2_O_3_, and ZnO nanoparticles were 81 ± 1, 41 ± 1, and 20.8 ± 0.2 m^2^/g, respectively.

Although some of the particles in Figure 1 appear as aggregates, it is difficult to infer the state of aggregation of particles from the TEM images. In acquiring the TEM images, samples of particles were dispersed in suspension and then dropped onto TEM grids and dried. This process inevitably leads to the appearance of aggregates as the sample dries; therefore, one must remain cautious about interpreting the images as indicative of aggregation in the dispersed phase. As described in “Materials and Methods,” particle stock solutions were sonicated for 5 min prior to preparing the final working concentrations; however, it is possible that some particle agglomeration occurs during the course of exposure to cells, even after dispersion with a sonicator.

**Figure 2:**
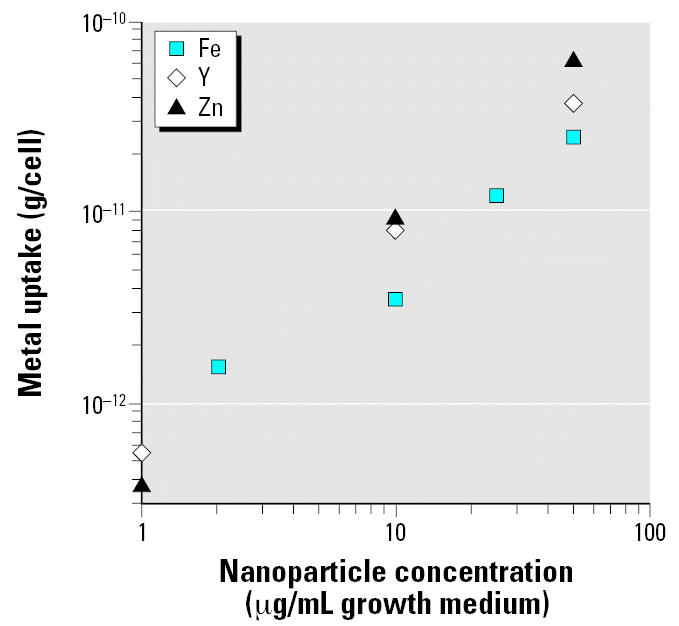
ICP-MS measurements of metal uptake by HAECs as a function of the metal oxide concentration in the extracellular solution for the three different types of nanoparticles.

ICP-MS measurements revealed that the amount of metal uptake correlated directly with the concentration of metal oxide in the extracellular solution for all three types of nanoparticles (Figure 2). These results indicate a clear dose-dependent uptake of the metal oxide nanoparticles by HAECs and show that nanoparticle concentration within the cell culture medium provides an accurate measure of the particle dose delivered to the cell surface.

### Thin-section TEM images of cells

**Figure 3:**
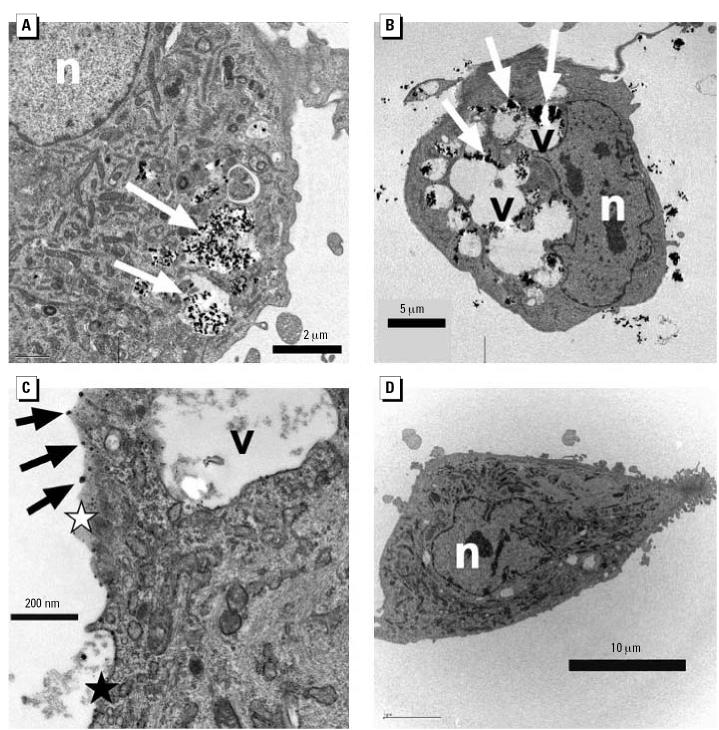
Thin-section TEM images of HAECs incubated with metal oxide nanoparticles. (*A*) Fe_2_O_3_. (*B*) Y_2_O_3_. (*C*) ZnO. (*D*) Control (no nanoparticles). Abbreviations: n, nucleus; v, vesicle; black star, normal continuous cell membrane; white star, region of possible cell membrane discontinuity. Arrows denote metal oxide particles or particulate matter.

Thin-section TEM images show that after 4 hr incubation Fe_2_O_3_ nanoparticles were incorporated into HAECs at all concentrations tested (0.001–50 μg/mL); the number of nanoparticles in a cell increased with concentration. Identification of Fe_2_O_3_ nanoparticles inside the cells was straightforward because the particles maintained their faceted feature. The Fe_2_O_3_ nanoparticles were often present within cytoplasmic vesicles (Figure 3A). No significant swelling was observed in the vesicles containing Fe_2_O_3_ nanoparticles.

Objects with high electron density were found in the thin-section TEM images of HAECs treated with Y_2_O_3_ nanoparticles. As with Fe_2_O_3_ nanoparticles, these objects were often present within cytoplasmic vesicles (Figure 3B), and their density increased with nanoparticle concentration. Although they did not have the characteristic morphology of the original Y_2_O_3_ nanoparticles (Figure 1B) and despite the lack of elemental analysis to confirm their chemical composition, these high electron density objects are presumably the products of the degradation of the Y_2_O_3_ nanoparticles, because no other elements of comparable electron density should be present in these thin sections and the objects were not observed in the control sample (Figure 3D). The vesicles containing these objects exhibited considerable swelling, particularly at the higher Y_2_O_3_ nanoparticle concentrations (Figure 3B).

Thin-section TEM images of HAECs treated with ZnO nanoparticles showed fewer intracellular vesicles (Figure 3C). High electron density particulate matter was observed in the vicinity of the cell membrane. Such particulate matter was not seen in the control (untreated) cells (Figure 3D). In some instances, apparent discontinuities in the cell membrane were observed in HAECs treated with ZnO nanoparticles.

### Effect of nanoparticles on HAEC inflammation and viability

**Figure 4:**
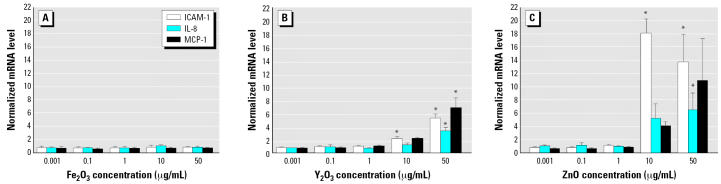
mRNA levels of the three inflammatory markers ICAM-1, IL-8, and MCP-1 in HAECs incubated for 4 hr with Fe_2_O_3_ (*A*), Y_2_O_3_ (*B*), or ZnO (*C*) nanoparticles. Each mRNA value was normalized to corresponding GAPDH value. Ratios relative to control cells (no nanoparticles) are shown; data are mean ± SE from three independent experiments run in duplicates. *Statistically significant mRNA level increase relative to control cells (*p* < 0.05).

Incubating HAECs for 4 hr with Fe_2_O_3_ nanoparticles failed to induce an increase in ICAM-1, IL-8, or MCP-1 mRNA levels relative to control cells (Figure 4A). For instance, the ICAM-1, IL-8, and MCP-1 mRNA levels of HAECs incubated with 50 μg/mL Fe_2_O_3_ nanoparticles were 0.8 ± 0.2, 0.8 ± 0.2, and 0.7 ± 0.1 times control values, respectively (*p* > 0.05). In contrast, 4-hr incubation with Y_2_O_3_ nanoparticles induced an increase in ICAM-1, IL-8, and MCP-1 mRNA levels. The effect of Y_2_O_3_ nanoparticles was concentration dependent, with a statistically significant increase at the two highest concentrations: 10 and 50 μg/mL (Figure 4B). At 50 μg/mL, Y_2_O_3_ nanoparticles increased ICAM-1, IL-8, and MCP-1 mRNA levels relative to control cells by 5.3 ± 0.6, 3.3 ± 0.5, and 6.8 ± 1.6 times, respectively (*p* < 0.05 for all three markers). At 10 μg/mL, the equivalent mRNA up-regulation values were 2.2 ± 0.3, 1.2 ± 0.3, and 2.2 ± 0.1 for ICAM-1, IL-8, and MCP-1, respectively (*p* < 0.05 for ICAM-1; *p* > 0.05 for MCP-1 and IL-8).

ZnO nanoparticles had the most striking effect on HAECs. At the two highest concentrations, ZnO nanoparticles provoked considerable cell death at the end of the 4-hr incubation period. This was unlike the Fe_2_O_3_ and Y_2_O_3_ nanoparticles, which did not lead to visible cell loss after 4 hr of incubation. At 50 μg/mL, cell loss after 4 hr ZnO incubation was approximately 50%; at 10 μg/mL, cell loss was approximately 20%. No cell loss was noted at the lower concentrations. An 8-hr incubation period did not result in additional cell loss. HAECs remaining attached following incubation with ZnO nanoparticles showed similar viability rates as assessed by trypan blue exclusion to those of control cells and cells exposed to the other two types of nanoparticles. More specifically, HAEC viability rates after 4-hr incubation were as follows (*n* = 3 and *p* > 0.05 relative to control in all cases): 88 ± 1% for control cells; 89 ± 1% and 82 ± 3% for ZnO nanoparticles at 10 and 50 μg/mL, respectively; 88 ± 2% and 88 ± 4% for Y_2_O_3_ particles at 10 and 50 μg/mL, respectively; and 88 ± 4% and 92 ± 3% for Fe_2_O_3_ particles at 10 and 50 μg/mL, respectively. Largely similar viability rates were obtained after 8 hr of incubation.

To assess the longer-term impact of ZnO nanoparticles on HAEC viability, cells were incubated with these particles at either 10 or 50 μg/mL for 4 hr, washed with PBS three times, and then maintained another 24 hr in cell culture medium free of nanoparticles. Although this protocol did not lead to a significant decrease in cell viability at 10 μg/mL (89 ± 2% vs. 90 ± 1% for control cells; *n* = 4, *p* > 0.05), viability significantly decreased at 50 μg/mL (71 ± 7%; *n* = 4, *p* < 0.05 relative to control). However, there are technical difficulties associated with the protocol. We found it very difficult to fully remove particles from the cell surface by washing, especially at the higher particle concentrations. Therefore, the particles that remained attached after washing may have continued to influence cell viability during the ensuing 24-hr period.

ZnO nanoparticles also elicited an inflammatory reaction in HAECs in a concentration-dependent manner (Figure 4C). At 50 μg/mL, ICAM-1, IL-8, and MCP-1 mRNA levels increased relative to control cells by 13.6 ± 4.1, 6.4 ± 2.5, and 10.8 ± 6.2 times, respectively. The equivalent values for 10 μg/mL were 17.9 ± 2.1, 5.2 ± 2.2, and 4.0 ± 0.6.

**Figure 5:**
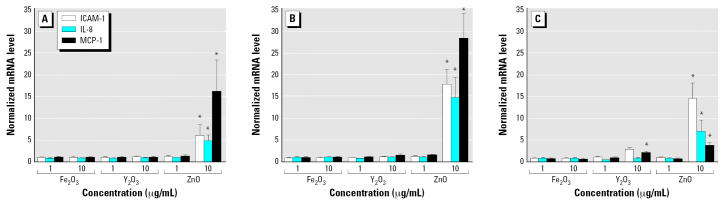
mRNA levels of the three inflammatory markers ICAM-1, IL-8, and MCP-1 in HAECs incubated with nanoparticles (1 and 10 μg/mL) for 1 hr (*A*), 2 hr (*B*), and 8 hr (*C*). Each mRNA value was normalized to corresponding GAPDH value. Ratios relative to control cells (no nanoparticles) are shown; data are mean ± SE from three independent experiments run in duplicates. *Statistically significant mRNA level increase relative to control cells (*p* < 0.05).

To elucidate the temporal evolution of transcriptional inflammatory changes induced by nanoparticles, we studied ICAM-1, IL-8, and MCP-1 mRNA levels at three additional time points: 1, 2, and 8 hr. Because the 4-hr data had demonstrated that the critical concentration threshold required for inducing an inflammatory response was between 1 and 10 μg/mL (Figure 4), these two concentrations were selected for the experiments at the additional time points. At the 1- and 2-hr time points, only ZnO nanoparticles at 10 μg/mL increased ICAM-1, IL-8, and MCP-1 mRNA levels significantly (Figure 5A, 5B). This increase persisted at the 8-hr time point and was also accompanied by a more modest increase in ICAM-1 and MCP-1 mRNA in HAECs incubated with Y_2_O_3_ nanoparticles at 10μg/mL (Figure 5C). Fe_2_O_3_ nanoparticles failed to elicit inflammation at any of the time points or concentrations tested.

Although ZnO nanoparticles altered the transcriptional expression of all three inflammatory markers, the dynamics of this increase were different for the different markers. Although ICAM-1 mRNA levels increased between the 1- and 2-hr time points and then remained at this elevated level, IL-8 and MCP-1 mRNA expression peaked at 2 hr and then decreased to considerably lower levels (though significantly higher than control levels) at the 4- and 8-hr time points.

**Figure 6:**
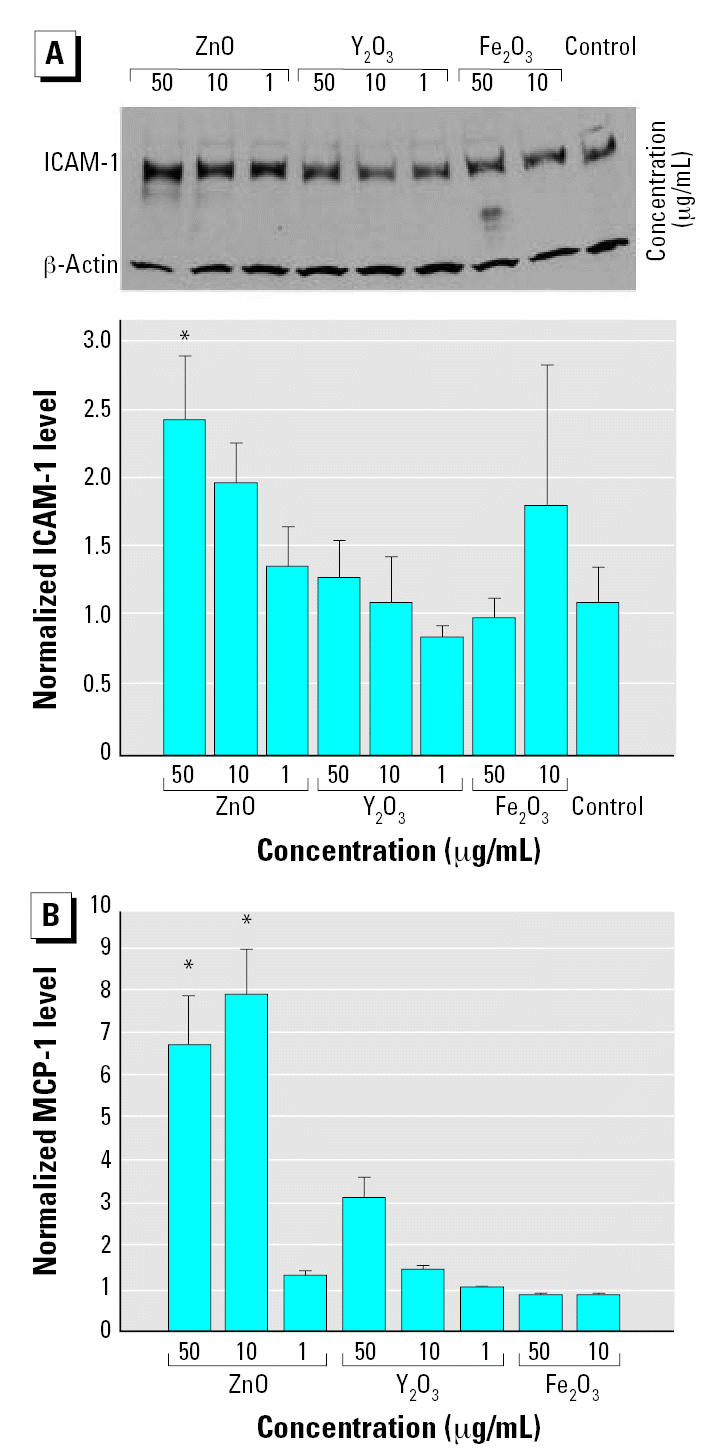
Protein levels of ICAM-1 (*A*) and MCP-1 (*B*) in HAECs incubated with nanoparticles (1, 10, and 50 μg/mL) for 4 hr. (*A*) Representative Western blot and densitometric analyses of ICAM-1 protein levels. ICAM-1 band densities are normalized to the internal control (β-actin); data are mean ± SE from 3–6 independent experiments. (*B*) ELISA results for MCP-1 protein levels relative to control cells (no nanoparticles); data are mean ± SE from 3–4 independent experiments. *Statistically significant protein level increase relative to control cells (*p* < 0.05).

In addition to the transcriptional response, the up-regulation of inflammatory markers by nanoparticles was also observed at the translational (protein) level. We used Western blot analysis to probe ICAM-1 protein levels in HAECs following 4-hr incubation with nanoparticles (Figure 6A). Fe_2_O_3_ and Y_2_O_3_ nanoparticles failed to increase ICAM-1 protein at any of the concentrations tested. On the other hand, ZnO nanoparticles at 10 and 50 μg/mL increased ICAM-1 protein levels relative to control cells by 1.8 ± 0.5 and 2.2 ± 0.6 times, respectively (*p* = 0.06 for 10 μg/mL and *p* < 0.05 for 50 μg/mL). We also measured MCP-1 protein concentration in cell culture supernatants of HAECs incubated with nanoparticles for 4 hr using ELISA (Figure 6B). In line with the mRNA results, Fe_2_O_3_ nanoparticles did not increase MCP-1 levels. At 10 and 50 μg/mL, Y_2_O_3_ nanoparticles increased MCP-1 protein levels relative to control cells by 1.5 ± 0.1 and 3.2 ± 0.5 times, respectively (*p* > 0.05 for both). Similar concentrations of ZnO nanoparticles increased MCP-1 concentrations by 8.0 ± 1.1 and 6.8 ± 1.2 times (*p* < 0.05 for both). A concentration of 1 μg/mL failed to evoke a significant increase in MCP-1 protein levels for all three types of nanoparticles.

### Role of released metals and impurities in HAEC inflammation

A question that arises is whether the inflammatory response is due at least in part to the release of reactive metal species from the nanoparticles. To address this issue, we performed limited experiments in which cell culture medium incubated for 4 hr with Fe_2_O_3_, Y_2_O_3_, or ZnO nanoparticles (at either 10 or 50 μg/mL) was centrifuged to remove most nanoparticles, and the supernatant was then added to HAECs for another 4 hr. For all three types of nanoparticles, ICAM-1, IL-8, and MCP-1 mRNA levels were virtually identical to those of control cells (data not shown). These findings suggest that the observed inflammatory response is due to the presence of the particles rather than released reactive metals.

We also determined the detailed metal composition of all three types of nanoparticles using ICP-MS. The results demonstrated that the synthesized nanoparticles were pure because concentrations for the vast majority of metals were below the detection limit for all three types of nanoparticles. In the few cases where trace metal impurities were detected, they were at concentrations on the order of 0.01 ppb (very close to the detection limit; data not shown).

## Discussion

The mechanisms governing the epidemiologic correlation between exposure to ultrafine particles and increased incidence of cardiovascular disease remain unknown. Because endothelial inflammation is a critical early event in vascular pathology, we studied the impact of nanoparticles on inflammation in HAECs. Our results demonstrated that direct and acute exposure of HAECs to Y_2_O_3_ or ZnO nanoparticles significantly up-regulates mRNA levels of the inflammatory markers IL-8, ICAM-1, and MCP-1, whereas Fe_2_O_3_ particles have no effect. The mRNA up-regulation is larger for ZnO than for Y_2_O_3_. Whenever it occurs, the inflammatory response does not initiate below a particle concentration of approximately 10 μg/mL and is dose-dependent above this threshold concentration. At the highest concentration (50 μg/mL), ZnO nanoparticles lead to considerable cell toxicity in addition to the pronounced inflammatory response.

A key finding of the present study is that metal oxide nanoparticle composition is a major determinant of propensity to induce inflammation in HAECs; however, the mechanistic basis of this observation remains unknown. The dependence of endothelial inflammation on nanoparticle composition is not attributable to differences in the ability of the different types of particles to access the cell surface or to get internalized within the intra-cellular space. Our results show that all three types of metal oxide nanoparticles are internalized within HAECs and that a given particle concentration in the cell culture medium leads to a related dose delivered to the cells. Therefore, it is more likely that nanoparticle type may influence the inflammatory response through a dependence of specific inflammation-signaling pathways on particle composition. Interestingly, HAEC inflammation appears to correlate inversely with nanoparticle-specific surface area, although this issue needs to be studied more systematically in future studies. Fe_2_O_3_ nanoparticles, which have the largest specific surface area among the three types of particles tested (as shown by BET measurements), fail to elicit inflammation, whereas ZnO nanoparticles have the smallest specific surface area and provoke the most pronounced inflammatory response.

We determined that the possible release of reactive metal species from the nanoparticles does not contribute significantly to the inflammatory response in HAECs. This result is consistent with previous findings that although nickel and cobalt ions elicit ICAM-1, E-selectin, IL-6, and IL-8 production in human umbilical vein endothelial cells (HUVECs) (Wagner et al. 1998), nickel nanoparticles have virtually no impact on HUVEC inflammation or toxicity (Peters et al. 2004). A recent study demonstrating that the release of soluble components is not responsible for the inflammatory properties of carbon black nanoparticles (Brown et al. 2000) further corroborates the notion that the impact of free metal release from nanoparticles on inflammation is minimal.

An obvious candidate pathway for nanoparticle-induced inflammation in HAECs is the production of reactive oxygen species (ROS). Exposure to nanoparticles is generally thought to elicit ROS generation and cellular oxidative stress (Donaldson et al. 2001; Nel et al. 2006); however, a recent report has suggested that cerium oxide and Y_2_O_3_ nanoparticles act as antioxidants in neurons (Schubert et al. 2006). In preliminary studies, we tested the impact of Fe_2_O_3_, Y_2_O_3_, and ZnO nanoparticles (1, 10, and 50 μg/mL for 1, 2, and 4 hr) on HAEC ROS levels using the fluorescent indicator 5-(and-6)-chloromethyl-2′,7′ dichlorodihydrofluorescein diacetate, acetyl ester (Invitrogen). Visual inspection of the images could not positively confirm the presence of ROS; quantification of these results proved difficult because of the high background fluorescence in the images. Further studies with more sensitive reagents are needed to definitively establish a link between ROS formation and the inflammatory responses that we observed, if in fact ROS is responsible. Reactive nitrogen species may also be involved in endothelial inflammation induced by nanoparticles. Interestingly, a recent study has demonstrated that exogenous Zn elicits an endothelial stress response that resembles that of nitric oxide-mediated nitrosative stress (Wiseman et al. 2006).

We focused on Fe_2_O_3_, Y_2_O_3_, and ZnO nanoparticles as examples of metal oxides that are associated with environmental and occupational exposures. Huggins et al. (2000) analyzed Standard Reference Material (SRM) urban and diesel engine particulate matter (National Institute of Standards and Technology, Gaithersburg, MD) for metal content. They found that Fe and Zn were the most abundant metals following potassium and calcium in both types of samples. Yttrium is widely used in catalysts and is also used as a host material for phosphors that are used in lighting and computer displays. The three metals also provide a range of properties of their oxides, especially reactivity and solubility in acidic environments that might be found in cytoplasmic vesicles in endothelial cells (Ollinger and Brunk 1995; Spragg et al. 1997). Future studies should test if differences in the propensity of different metal oxide nanoparticles to induce inflammation may relate to the reactivity of the metal oxides in acidic environments.

Recent studies have focused on using nanoparticles for targeted drug delivery to cells and for visualizing intracellular processes (Panyam and Labhasetwar 2003; Pitsillides et al. 2003). These studies investigated nanoparticle uptake by cells but did not address in detail the impact of nanoparticles on cellular pathology. Previous studies have demonstrated that exposure to ultrafine particles induces inflammation in pulmonary epithelial cells and within the lung in general (Dagher et al. 2005; Schaumann et al. 2004). However, very few studies have addressed the impact of ultrafine particles on vascular cells. *In vivo,* inhalation of particulate matter–laden air has been shown to induce release of endothelin-2 in rats (Elder et al. 2004). *In vitro,* incubation of HUVECs with particulate matter collected from air sampling has been reported to increase E-selectin production (Alfaro-Moreno et al. 2002). More recently, Peters et al. (2004) reported that direct exposure of dermal microvascular endothelial cells to metal nanoparticles decreases cellular viability and induces IL-8 expression. Interestingly, the response was dependent on nanoparticle composition: it was more pronounced for cobalt particles than for either silicon dioxide or titanium dioxide particles and was absent for nickel and polyvinyl chloride particles. Finally, in a recent study, Yamawaki and Iwai (2006) demonstrated that carbon black nanoparticles (at concentrations of up to 100 μg/mL) alter HUVEC morphology and increase MCP-1 protein levels in a dose-dependent fashion, similar to the ZnO and Y_2_O_3_ nanoparticles in the present study.

In the present study, we examined the effect of a wide range of nanoparticle concentrations on HAEC inflammation. The fraction of inhaled particles that ultimately cross the pulmonary epithelial barrier into the cardiovascular system is not definitively known; therefore, particle concentrations to which endothelial cells are exposed *in vivo* remain to be determined. Some animal studies suggest that particles translocating to extrapulmonary organs account for a small fraction (< 1%) of those deposited in the lungs (Kreyling et al. 2002). On the other hand, other studies have reported that approximately 10–15% of inhaled titanium oxide nanoparticles end up within the vascular compartment (Geiser et al. 2005). In a reported case of human Zn fume fever (Noel and Ruthman 1988), the serum Zn level was elevated from 1.1 to 1.6 μg/mL. If the excess Zn were in the form of ZnO particles, the increase would correspond to a ZnO concentration of 0.5 μg/mL in the bloodstream. Although this value falls below the threshold required to induce inflammation reported here (~ 10 μg/mL), it is essential to recognize that, in the present study, we focused exclusively on acute exposure (1–8 hr). Although the results of acute studies are critical in light of the observation that many of the cardiovascular events correlated with particulate matter exposure occur within a few hours of exposure (Peters et al. 2001), inflammation induced by inhaled/translocated particles likely persists for years. Therefore, particles that are identified in acute studies to mediate an inflammatory response can subsequently be tested at lower doses to evaluate their chronic effect.

In the present study we focused on nanoparticle interactions with endothelium under static conditions. Endothelial cells *in vivo* are constantly exposed to flow, and there is mounting evidence that fluid mechanical forces regulate endothelial inflammation. Flow is also expected to alter the rate of delivery of nanoparticles to the endothelial cell surface and the particle interaction time with the cell surface. Therefore, future studies performed under flow promise to provide a more physiologically relevant understanding of endothelial cell inflammation induced by nanoparticles. Finally, it would be important to establish if the present results obtained from endothelial monolayers in culture also apply to the *in vivo* environment. Animal studies of the impact of nanoparticles on vascular inflammation should help address this question.
